# Gastric duplication cyst causing hypergastrinemia in an infant

**DOI:** 10.1002/jpr3.70176

**Published:** 2026-04-06

**Authors:** Nathan Bryan, Ian Leibowitz

**Affiliations:** ^1^ Division of Gastroenterology Children's National Hospital Washington DC USA

**Keywords:** esophageal necrosis, malnutrition, peptic ulcer disease, vomiting

## Abstract

Gastric duplication cysts are rare congenital anomalies, with fewer than 10% occurring in the stomach. We report a 14‐month‐old girl presenting with failure to thrive, recurrent emesis, hematemesis, and severe duodenitis with ulceration. Laboratory evaluation revealed marked hypergastrinemia (1781 pg/mL), and initial imaging was unrevealing. Persistent symptoms prompted repeat imaging, which identified a 3.7 × 5.2 cm gastric duplication cyst causing gastric outlet obstruction. Surgical resection confirmed gastric mucosa within the cyst, and postoperative gastrin levels normalized. This case represents one of very few reports linking gastric duplication cysts to hypergastrinemia, likely due to isolation of functional gastric mucosa within a closed lumen, resulting in excessive gastrin secretion and peptic injury. The case underscores the importance of considering duplication cysts in pediatric patients with poor growth and the utility of repeat imaging when symptoms persist despite negative initial studies.

## 1 INTRODUCTION

Enteric duplication cysts are rare congenital anomalies, less than 10% of which are found in the stomach. They present in a variety of ways, including vomiting, abdominal pain, or palpable abdominal mass. Less common features, which this case highlights, include malnutrition and hematemesis.^1^ Cyst histology drives physiology as enzyme and hormone secretion is based on heterotopic tissue. Gastrin‐secreting cysts cause hypergastrinemia because trapped, functioning gastric mucosa is exposed to minimal acidity, stimulating G‐cell activity without somatostatin‐mediated feedback.^2^

## 2 CASE REPORT

**Figure 1:**
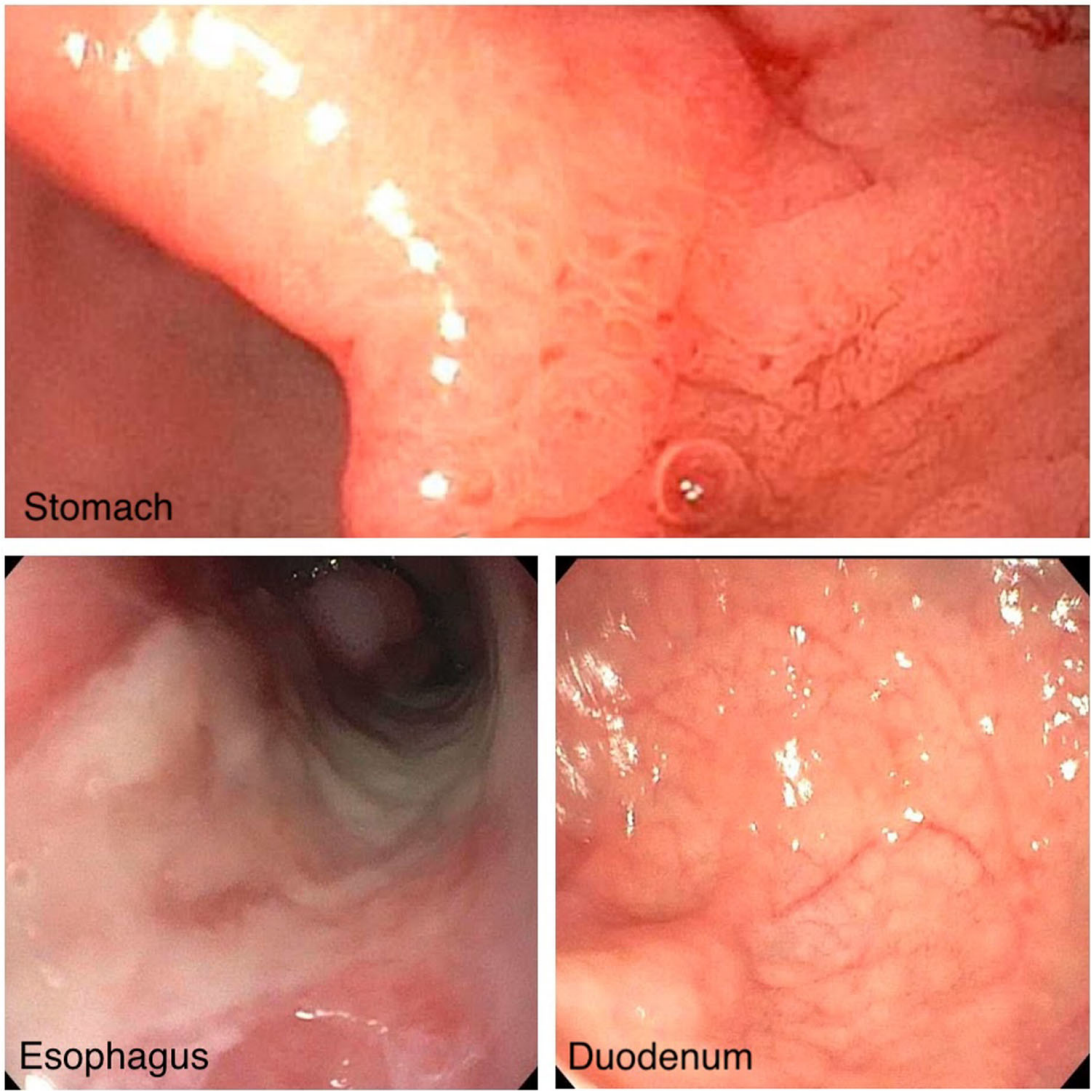
EGD #1 LA Grade C in the esophagus, gastric erosions, and normal duodenum. EGD, esophagogastroduodenoscopy; LA, Los Angeles.

**Figure 2:**
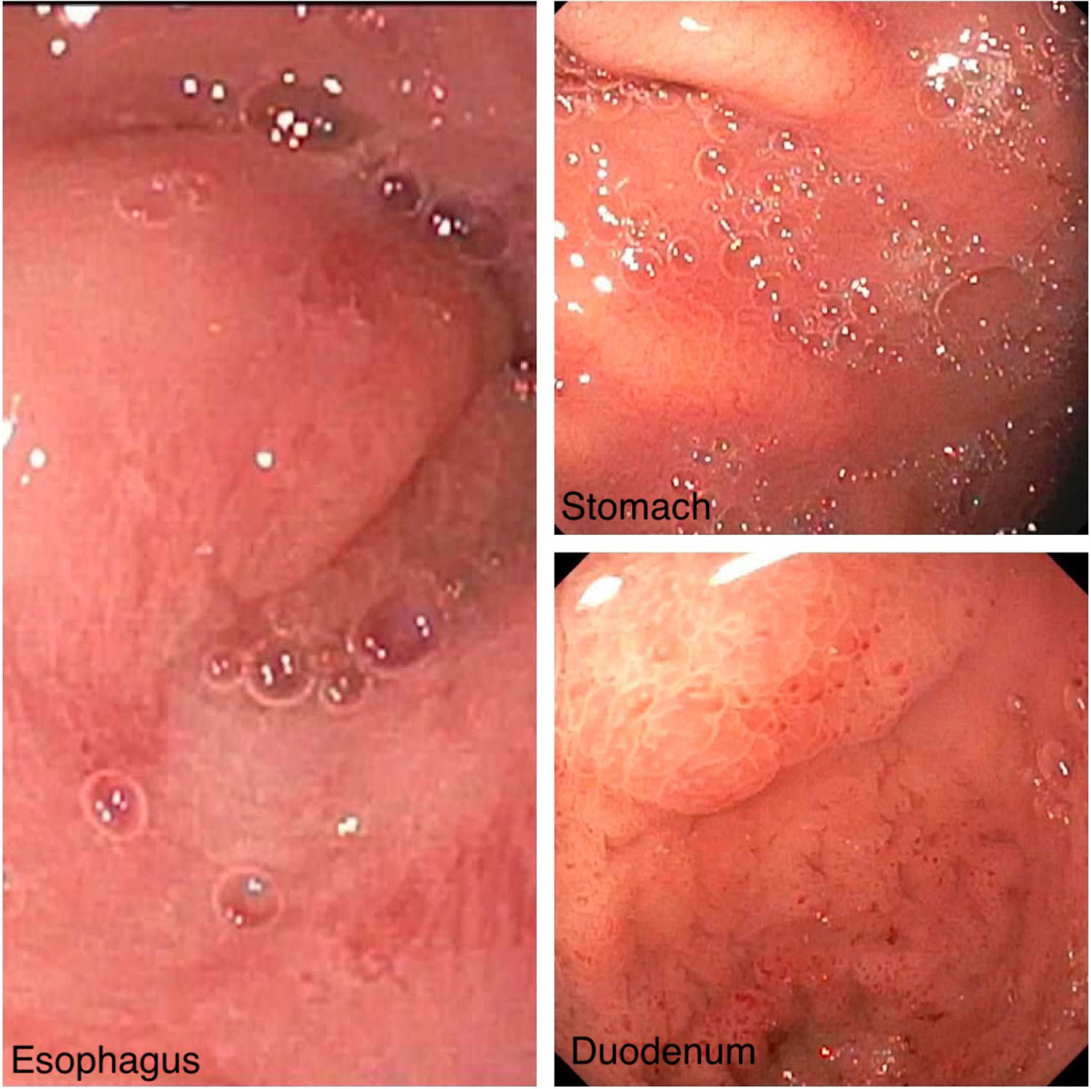
EGD #2 Healing esophagus, gastric erosions, and now significant ulceration in the duodenum. EGD, esophagogastroduodenoscopy.

**Figure 3:**
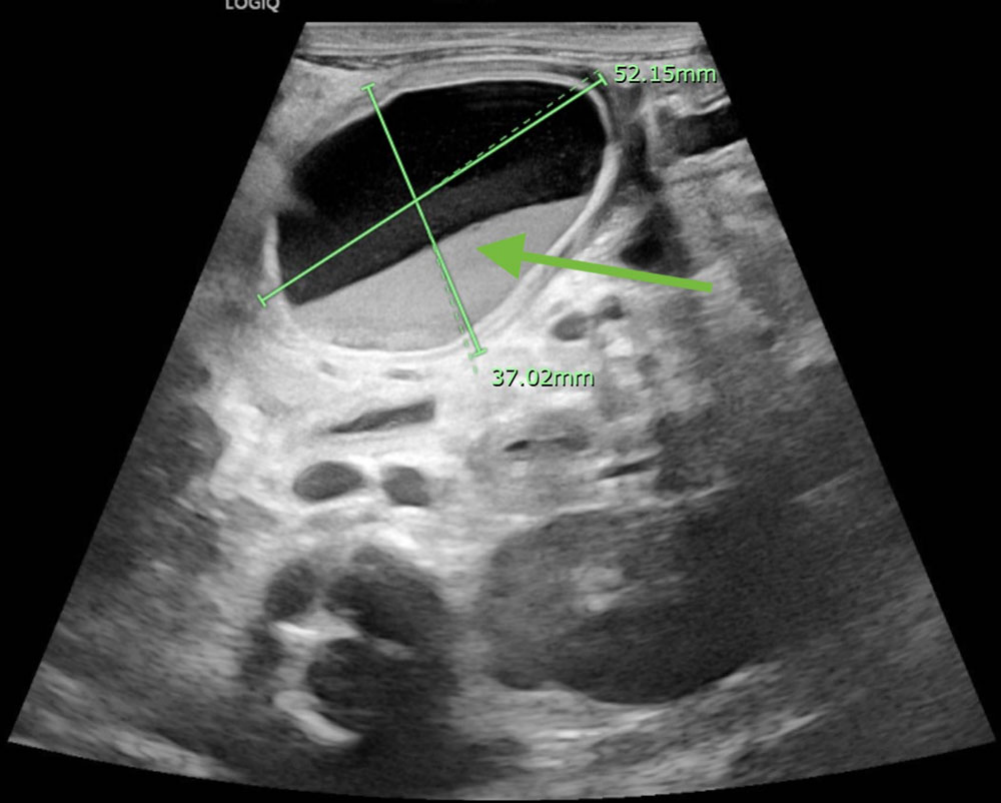
Ultrasound #2 5.2 × 3.7 cm cystic structure with fluid debris (arrow) and double mucosa sign.

A 14‐month‐old girl presented to the emergency room with concerns for failure to thrive. She had a history of recurrent emesis and abdominal distension. Her weight was less than the first percentile (7 kg, body mass index 14.5), a notable decline from her 26th percentile birthweight. Her physical exam was notable for mild abdominal distention and tenderness. Her vital signs were within normal limits for her age. Laboratory workup was notable for a low albumin of 2.9 g/dL, a hemoglobin of 9.8 g/dL, aspartate aminotransferase (AST) of 135 IU/L, and alanine transaminase (ALT) of 136 IU/L. The remainder of her laboratory values, including complete blood count, metabolic profile, lipase, bilirubin, and urinalysis, were within normal limits. An upper gastrointestinal series and abdominal ultrasound were performed and found to be normal. Possibilities considered for her malnutrition included malabsorptive conditions like celiac disease, pancreatic insufficiency, and protein‐losing enteropathy but eventually attributed to insufficient caloric intake, and a nasogastric tube was placed for supplemental feeding. Three days after discharge, she re‐presented with hematemesis and a hemoglobin of 6.8 gm/dL. An upper endoscopy revealed significant ulceration, Los Angeles (LA) Score C, with esophageal necrosis confirmed by histology (Figure 1). On histology, her stomach had gastritis, and the duodenum appeared normal. Possible etiologies for esophagitis suggested included caustic ingestion, tube‐related trauma, or severe peptic injury. She was started on a proton pump inhibitor (PPI) for 6 months and was discharged home. When repeating a surveillance endoscopy 2 months later, duodenitis with ulcers was found (Figure 2). Histopathology confirmed these findings; staining for *Helicobacter pylori* was negative, and the family denied nonsteroidal anti‐inflammatory drug use. A fasting gastrin level was elevated at 1781 (reference range in PPI‐exposed patients: 48–487).^3^ A differential diagnosis of elevated gastrin levels included gastrinoma, infection, or gastric outlet obstruction. Two weeks later, she was re‐admitted due to persistent emesis. An upper gastrointestinal series at this time showed complete gastric outlet obstruction. An abdominal ultrasound now revealed a gastric duplication cyst measuring 3.7 × 5.2 cm with double mucosa sign located near but separate from the gastric antrum (Figure 3). Retrospective review of her initial imaging did not reveal a previously missed cyst. With surgical consultation, the duplication cyst was resected, and pathology confirmed gastric mucosa. She recovered quickly and was discharged home tolerating full feeds. One week later, gastrin levels had reduced to 17 and her weight improved to the 33rd percentile. AST and ALT levels also normalized, which may suggest the cyst had hepatic compression as well. She was maintained on a PPI for the next 12 months. An endoscopy 9 months after surgery showed no abnormalities. A repeated ultrasound 1 year after resection showed no evidence of residual or recurrent cystic lesion.

## 3 DISCUSSION

Gastric duplication cysts are typically seen on ultrasound or other imaging modalities, but diagnosis can be missed until the cyst enlarges.^4^ In our case, though the cyst was symptomatic, it was not initially detected with repeated radiologic studies. Cysts may progressively enlarge over weeks to months, although the exact rate of growth is not well documented.^2^ While prenatal diagnosis is often difficult, and standard prenatal ultrasound identifies only about 20–30% of duplications,^5^ postnatal ultrasound is likely more sensitive. Defined sensitivity and specificity rates of ultrasound for detecting duplication cysts are lacking, given their rarity and the large range in locations and presentations. Some reports estimate that ultrasound can identify abdominal cysts with accuracy rates of 80–95%.^6^ Both computed tomography and magnetic resonance imaging can detect cysts, but ultrasound remains the modality of choice due to radiation and sedation requirements and is generally reserved for surgical planning.^2^ When performing an ultrasound, special attention is required not to overlook or mistake small cysts near bowel or the gallbladder. Even in the absence of gastric outlet obstruction or growth faltering, incidental duplication cysts should be removed due to increased risk of malignant transformation.^7^ With surgical resection, there are rarely complications or recurrence.^8^

This case represents the fourth reported pediatric case of a gastric duplication cyst associated with hypergastrinemia.^7^, ^8^, ^9^ Tanaka et al. and Stephen et al. both highlight hypergastrinemia with peptic ulceration, whereas Youssef et al. describe a cyst presenting primarily with massive acute gastrointestinal hemorrhage rather than documented hypergastrinemia. There have been no reports of PPIs perpetuating gastric duplication cysts; however, it is possible these medications may mask some of the symptoms such as pain. PPIs limit pain primarily through acid suppression, which can be elevated when duplication cysts contain gastrin‐secreting heterotopic tissue.

## 4 CONCLUSION

This case serves as a reminder to consider gastric duplication cysts when encountering a patient presenting with ongoing malnutrition, duodenitis, vomiting, and severe esophageal injury. As early imaging may miss growing cysts, in the presence of ongoing symptoms, it is important to reimage to identify the cause.

## CONFLICT OF INTEREST STATEMENT

The authors declare no conflict of interest.

## ETHICS STATEMENT

Informed consent was obtained regarding the collection and publication of patient information for this case.
